# Extracellular vesicles mediated proinflammatory macrophage phenotype induced by radiotherapy in cervical cancer

**DOI:** 10.1186/s12885-022-09194-z

**Published:** 2022-01-21

**Authors:** Junli Ren, Lili Li, Baofeng Yu, Enwei Xu, Naiping Sun, Xiaoning Li, Zihan Xing, Xiaodong Han, Yaqin Cui, Xiaoyan Wang, Xiaoxue Zhang, Guoliang Wang

**Affiliations:** 1 Department of Radiotherapy Abdominopelvic, Shanxi Cancer Hospital, Taiyuan, 030013 Shanxi China; 2 Department of Biochemistry and Molecular biology, Shanxi Medical University, Taiyuan, 030001 Shanxi China; 3 Department of Pathology, Shanxi Cancer Hospital, Taiyuan, 030013 Shanxi China; 4 Department of Gynecology, Shanxi Cancer Hospital, Taiyuan, 030013 Shanxi China; 5 Department of Pediatric Surgery, Xiang’an Hospital of Xiamen University, Xiamen, 350213 Fujian China; 6 National Center for Children’s Health (NCCH), Beijing Pediatric Research Institute, Beijing Children’s Hospital, Capital Medical University, Beijing, 100045 China

**Keywords:** Extracellular vesicle, Macrophage, Radiotherapy, Cervical cancer

## Abstract

**Background:**

Radiotherapy is a highly effective treatment for cervical cancer. Recent studies focused on the radiotherapy induced anti-tumor immunity. Whether tumor-derived extracellular vesicles (EVs) play roles in radiotherapy induced tumor associated macrophage (TAM) polarization remains unclear.

**Materials and Methods:**

This study analysed the phenotype of macrophages in cancer tissue and peripheral blood of cervical cancer patients using flow cytometry analysis. The role of EVs from plasma of post-irradiated patients on M2-like transformed macrophages was assessed. The M1- and M2-like macrophages were assessed by expression of cell surface markers (CCR7, CD163) and intracellular cytokines (IL-10, TNFα and iNOS). The capacity of phagocytosis was assessed by PD-1 expression and phagocytosis of pHrodo Red E. coli bioparticles.

**Results:**

Our results demonstrated that radiotherapy of cervical cancer induced an increase in the number of TAMs and a change in their subtype from the M2-like to the M1-like phenotype (increased expression of CCR7 and decreased expression of CD163). The EVs from plasma of post-irradiated patients facilitated the M2-like to the M1-like phenotype transition (increased expression of CCR7, TNFα and iNOS, and decreased expression of CD163 and IL-10) and increased capacity of phagocytosis (decreased PD-1 expression and increased phagocytosis of pHrodo Red E. coli bioparticles).

**Conclusions:**

Our data demonstrated that irradiation in cervical cancer patients facilitated a proinflammatory macrophage phenotype which could eventually able to mediate anti-tumor immune responses. Our findings highlight the importance of EV in the crosstalk of tumor cells and TAM upon irradiation, which potentially leading to an increased inflammatory response to cancer lesions.

**Supplementary Information:**

The online version contains supplementary material available at 10.1186/s12885-022-09194-z.

## Introduction

Cervical cancer, which was mainly caused by carcinogenic human papillomavirus types, continues to be a major public health problem affecting middle-aged women [1, 2]. Although cytological screening [3, 4] and the use of vaccines against human papillomavirus [5, 6] have led to a major decline in cancer burden in several resource-rich countries, cervical cancer remains to be the most commonly diagnosed cancer and the predominant cause of cancer mortality in resource-poor countries, especially in sub-Saharan Africa and southeast Asia [1, 2].

Radiotherapy is a highly effective treatment for cervical cancer, even for patients with advanced stages [7]. Radiotherapy can result in direct cancer cell death by inducing DNA double strand break [8]. Recent studies focused on the radiotherapy induced anti-tumor immunity generated by various activating and/or inhibiting factors [9, 10]. By radiotherapy induced immunogenicity cell death, the damage-associated molecular patterns from tumor cells can induce an effective anti-tumor immune response [11]. Macrophages are one of the most abundant immune cell subsets in tumor tissue and play a key role in tumor progression and metastasis [12, 13]. In the tumor microenvironment, tumor-associated macrophages (TAM) display high plasticity upon immunological stimuli [13, 14]. The M1-like and M2-like macrophages, as two extreme phenotypes of macrophages, can promote or inhibit anti-tumor immune response, respectively [15]. Therefore, macrophage can be used as an attractive target for anti-tumor therapy, through proper reprogramming with enhanced antitumor features.

More recently, tumor-derived extracellular vesicles (EVs) have been reported can reprogram tumor-infiltrating lymphocytes [16]. The molecular cargos in EV, as small signaling molecules, can be transferred to other cell type to modulate cell functions [17, 18]. EVs isolated from the plasma of cancer patients have also been reported to have immune-regulatory activities [19]. It is not clear whether tumor cell derived EVs have different regulatory effects on macrophage before and after radiotherapy, especially in cervical cancer patients. In the present study, we compared the phenotype and function of macrophages in tissue of cervical cancer before and after radiotherapy. The regulatory effects of EVs from cervical cancer patients after radiotherapy on peripheral macrophages were also assessed.

## Materials and Methods

### Patients and sampling

This study recruited 12 cervical cancer patients (47.5 ± 7.8 years) at stages IB1- IB2 who received radical radiotherapy at Shanxi Provincial Cancer Hospital. Patients were identified through hospital registry systems and histopathological examination. Radiotherapy procedures include the pelvic external-beam radiotherapy and brachytherapy. The pelvic external-beam radiotherapy was performed at 1.8-2.0 Gy per fraction, for a total of 25 fractions and 45-50 Gy (intensity-modulated radiotherapy, Varian Medical Systems). The high-dose rate brachytherapy was performed, after 20 fractions of pelvic external-beam radiotherapy, at 7 Gy per fraction (for a week) and 4 fractions in total (Nucletron-Fletcher systems). The cancer biopsy and peripheral blood specimens of patients were collected before radiotherapy and after the first fraction of brachytherapy (within 3 days, before the next fraction of brachytherapy). Peripheral blood specimens were also collected from 6 adult healthy donors (46.0 ± 4.3 years). This study was performed according to the Declaration of Helsinki and approved by Shanxi Provincial Cancer Hospital Ethics Committee (approval number 2016015). Informed consent was obtained from all subjects (all subjects were older than 18 years in this study).

### Immunohistochemistry

The cancer tissue samples were fixed with formalin and embedded with paraffin. The expression of CD68 in TAMs was examined using immunohistochemistry on 5 μm thick whole mount tissue sections, and a Leica Bond MAX auto-stainer was used. The slides were dewaxed and antigenicity was retrieved using the Leica antigen retrieval solution, then the slides were incubated with the mouse monoclonal anti-human CD68 antibody (clone KP1, ZSGB-Bio) for 30 min. The staining was visualized using a diaminobenzidine system after secondary antibody incubation. The number of membranous CD68 positive cells in the whole tumor tissue sample was calculated and at least five randomly selected high power fields (400×) were examined. Original Hematoxylin-Eosin staining slices from the pathology archive were also reanalyzed to assess the presence and degree of TAMs.

### Isolation of single cells from cancer tissue and flow cytometry

Fresh biopsy of cervical cancer tissue was washed with cold RPMI 1640 medium, then the tissue was minced using razor blades and mechanically homogenized in cold RPMI 1640 medium containing 2.5% fetal bovine serum [20]. The suspensions were filtered using a 70μm cell strainer (BD Biosciences) and samples were stained immediately for phenotype analysis by flow cytometry. All steps were completed within 2 hours. Cell surface marker and intracellular cytokine staining for flow cytometry analysis were performed as described previously [21, 22]. Live/dead discrimination was performed with fixable viability dye (Zombie Red, BioLegend). The fluorescent antibodies used in flow cytometry analysis were listed in Table 1.

**Table 1:** The fluorescent antibodies used in flow cytometry analysis

Antibody	Clone (source)	Isotype
CD163	GHI/61 (Biolegend)	IgG1
IL-10	JES3-9D7 (Biolegend)	IgG1
PD-1	NAT105 (Biolegend)	IgG1
CD68	FA-11 (Biolegend)	IgG2a
CD14	63D3 (Biolegend)	IgG1
CD45	HI30 (Biolegend)	IgG1
CCR7	150503 (BD Biosciences)	IgG2a
iNOS	Clone 6 (BD Biosciences)	IgG2a
CD9	M-L13 (BD Biosciences)	IgG1
TNFα	MAb11 (BD Biosciences)	IgG1
CD11b	M1/70 (BD Biosciences)	IgG2b

### Isolation of EVs

Fresh peripheral blood specimens were centrifuged at 1,000 × g for 10 min at room temperature and then centrifuged at 2,500 × g for 15 min at room temperature to obtain platelet-free plasma. The platelet-free plasma was diluted 1:1 in PBS and EVs were isolated using the Total Exosome Isolation Kit (from plasma) (ThermoFisher Scientific) according to the manufacturer’s instruction, as our previously described [22]. Briefly, 0.2 volumes of Exosome Precipitation Reagent (from plasma) was added into the diluted plasma samples and incubated at room temperature for 10 min. After centrifuged at 10,000 × g for 5 min at room temperature, the supernatant was carefully aspirated and the precipitate (EVs) was resuspended with PBS. The EV solution was used immediately or stored in aliquots at -70 °C.

### Characterization of EVs

Morphological examination of isolated EVs was done using transmission electron microscope. The EVs fixed with 4% paraformaldehyde were loaded onto a 300 mesh copper grid, and then stained with 2% phosphotungstic acid for 1-2 min and dried by an electric incandescent lamp for 10 minutes. Data were acquired using a transmission electron microscope (JEOL JEM-2100) at an accelerating voltage of 160 KV. The number and size of EVs were determined through nanoparticle tracking analysis by a NanoSight NS300 instrument (Malvern Instruments, United Kingdom), as our previously described [22]. For optimal results, the EV solution was adjusted to obtain ~50 microvesicles per field of view. Data was analyzed by NTA 3.0 software (Malvern Instruments). Based on the surface marker CD9 and the membrane permeability of carboxyfluorescein diacetate succinimidyl ester (CFSE), EVs were also characterized by immunofluorescence staining. CFSE (25 μg/ml) and PE-anti-CD9 (10 μg/ml) were added into platelet-free plasma samples for 2 h at room temperature. Then plasma EVs were purified using Total Exosome Isolation Kit (from plasma) and resuspended with 20 μl PBS. Fluorescent stained EVs were smeared on glass slide and visualized using a laser-scanning confocal microscope (TCS SP8 STED, Leica, magnification 63×10). Considering that the Total Exosome Isolation reagent also precipitates free CFSE and labeled antibodies [23], fluorescent stained EVs from fetal bovine serum (FBS) were also visualized as the negative control for anti-human CD9. The total protein of EVs were extracted and characterized by SDS-PAGE protein separation, Coomassie Blue staining (significant differences in albumin distribution) and Western-blotting for EV markers (CD9 and TSG101) and non-EV marker (ApoA1) were performed, according to our previously described methods [24]. An additional Word file shows the original Coomassie Blue staining figure and Western-blotting figures in more detail (see Additional file 1).

### Macrophage differentiation

Peripheral blood mononuclear cells were obtained by Ficoll-Plaque density gradient centrifugation and seeded at a concentration of 2×10^6^/well in RPMI 1640 medium in 12-well plates. Monocytes were isolated using the ability of monocytes to adhere to non-tissue culture treated plastic culture dishes. Attached cells were cultivated in RPMI 1640 medium with Glutamax (Thermo Fisher Scientific) supplemented with 100 ng/mL macrophage colony stimulating factor (M-CSF, Thermo Fisher Scientific), 10% fetal bovine serum, 100 U/mL penicillin, 100 U/mL streptomycin and 0.25 μg/mL fungizone at 37°C. Cells were cultivated for 7 days with medium change per 48 hours. To obtain M2-polarized macrophages, cells were stimulated with 20 ng/mL interleukin (IL)-4 (BioLegend) and 20 ng/mL IL-13 (BioLegend) for 48 hours. The M2-polarized macrophages were co-cultured with EVs (isolated from equal volume of plasma) from patients before and after radiotherapy for 24 hours, and then the cell surface marker and intracellular cytokine were analyzed using flow cytometry. The EV concentrations (particle number) used in macrophage differentiation and phagocytosis were 5 times higher than that in plasma, or at indicated concentrations. The fluorescence antibodies used for flow cytometry analysis were listed in Table 1.

### Macrophage phagocytosis

The IL-4+IL-13 stimulated peripheral blood mononuclear cells were first co-cultured with EVs from patients before and after radiotherapy for 24 hours, and then resuspended in 100 μL pHrodo Red E. coli bioparticles (Thermo Fisher Scientific) and incubated at 37°C for 2 hours. The leucocytes were washed and stained for flow cytometry. The macrophage phagocytosis experiment on EVs was performed through incubating the EVs (labeled with CFSE) with the IL-4+IL-13 stimulated leucocytes at 37°C for 2 hours, after co-cultured with EVs (unlabeled) from patients before and after radiotherapy for 24 hours. The macrophages (CD45^+^, CD11b^+^ and CD14^+^ cells) that were PE or CFSE high were considered to be phagocytosing.

### Statistics

Wilcoxon matched-pairs signed rank test were used in comparison between two paired groups. Mann Whitney U test was used in comparison between two independent groups. A bilateral p value of less than 0.05 was considered as statistically significant. Statistical analysis was performed using GraphPad Prism 8.0.

## Results

### Increased TAMs in tissue of cervical cancer after radiotherapy

**Fig. 1:**
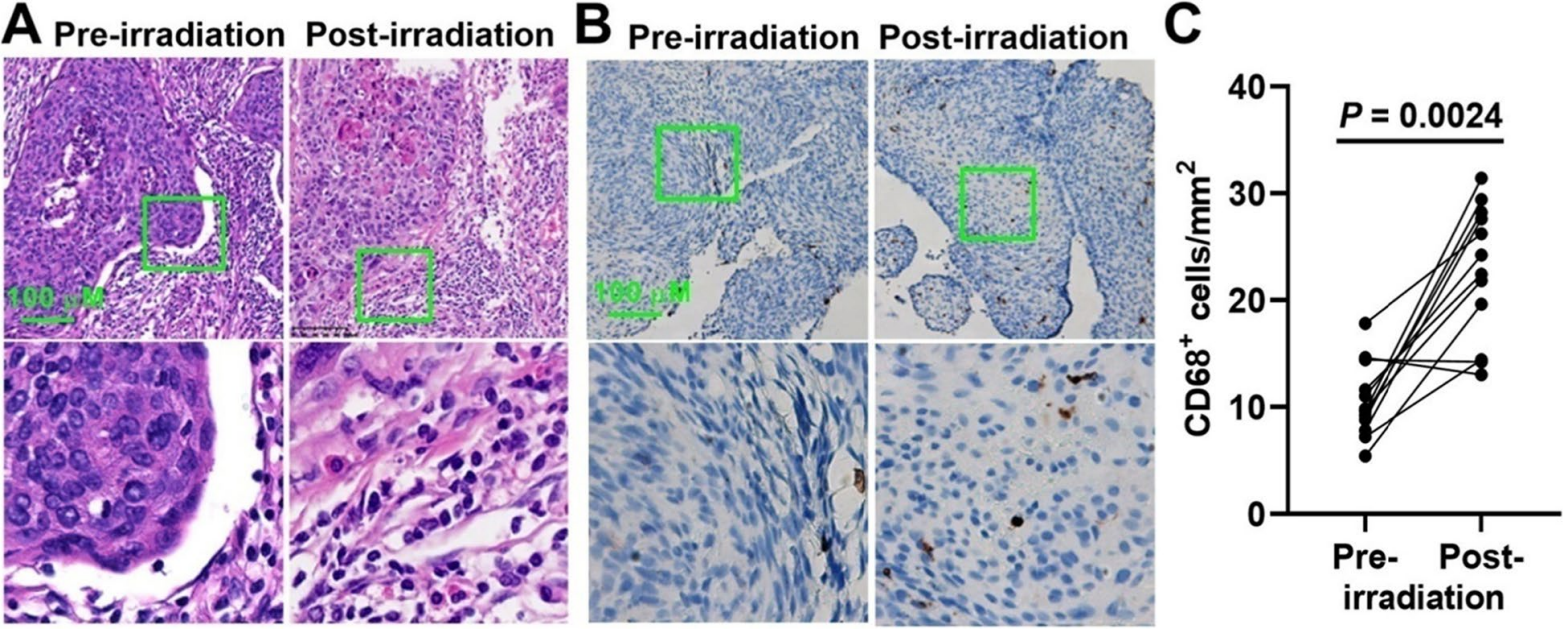
Immunohistochemical staining for tumor-associated macrophages (TAM) in cervical cancer tissue. The biopsies of cervical cancer patients were collected before and after radiotherapy. The cancer samples were fixed with formalin and embedded with paraffin. **A**, representative Hematoxylin-Eosin staining images. Images below were magnified 200×. **B**, representative images for CD68 staining. Images below were magnified 200×. **C**, the number of membranous CD68 positive cells was calculated in at least five randomly selected high power fields (400×). *P* value was calculated by Wilcoxon matched-pairs signed rank test

The presence and degree of TAMs in tissue of cervical cancer was first assessed before and after radiotherapy. As was shown in Fig. 1A, there seemed to be more TAM-like cells in cancer tissue after radiotherapy than cancer tissue before radiotherapy. Then we performed macrophage specific CD68 staining on tumor tissues to further clarify whether the amount of TAMs was changed after radiotherapy. Results show that the TAMs in cancer tissue significantly increased after radiotherapy, as shown in Fig. 1B and C.

### TAMs in tissue of cervical cancer showed an enhanced M1-like phenotype after radiotherapy

**Fig. 2:**
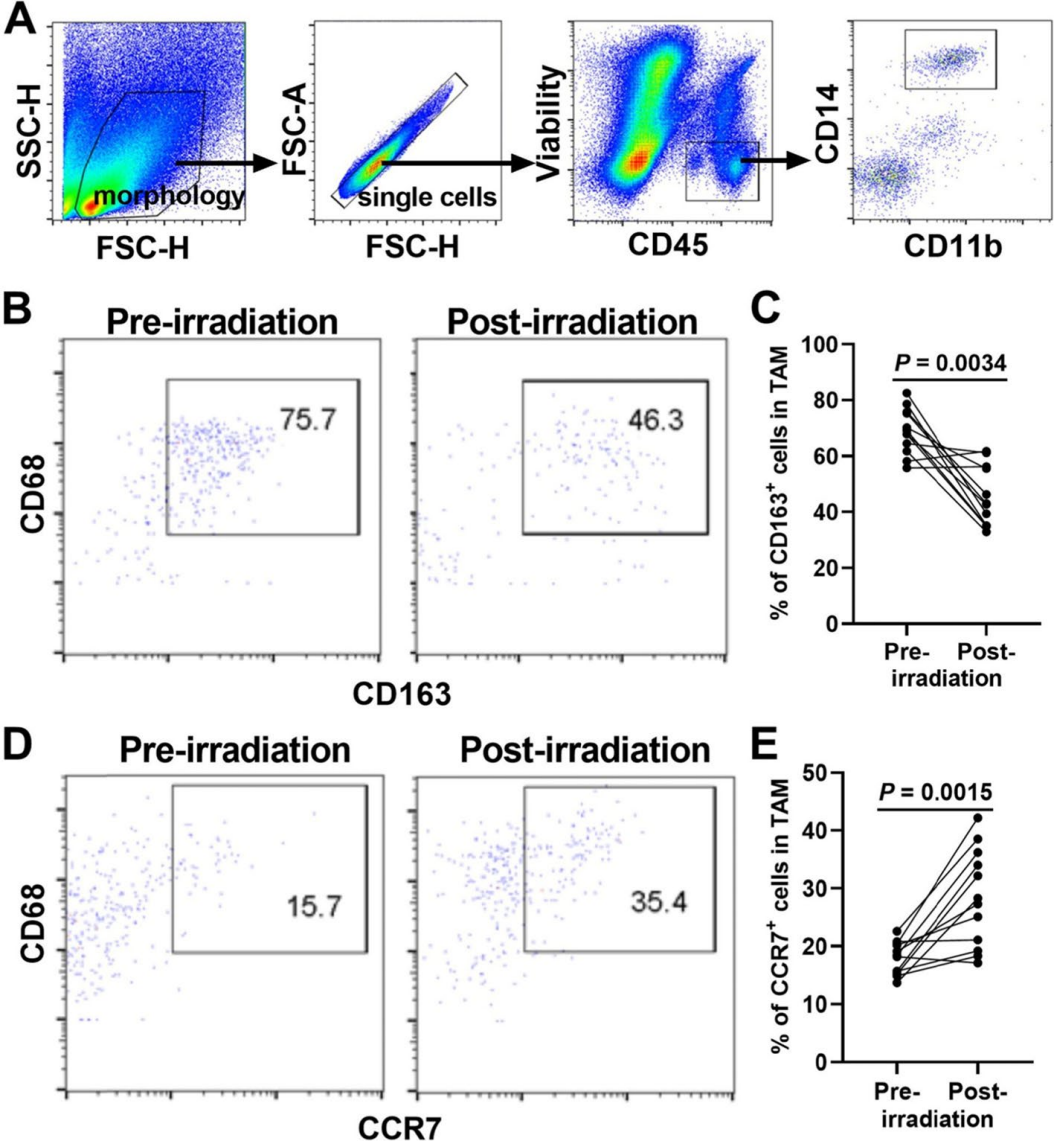
Flow cytometry analysis for TAMs in cervical cancer tissue. Fresh biopsies of cervical cancer tissue were minced and stained immediately for phenotype analysis by flow cytometry. **A**, representative example of the gating strategy for macrophages isolated from viable CD45^+^ mononuclear cells of cervical cancer patients. Representative scatter diagrams (**B**) and histograms (**C**) of CD68^+^CD163^+^ TAMs in cervical cancer tissue before and after radiotherapy. Representative scatter diagrams (**D**) and histograms (**E**) of CD68^+^CCR7^+^ TAMs in cervical cancer tissue before and after radiotherapy. *P* value was calculated by Wilcoxon matched-pairs signed rank test

Next, we explored the phenotype of tissue TAMs through comparing them in biopsies from the same cervical cancer patients before and after radiotherapy. The cervical cancer tissue was homogenized and the cell surface markers were assessed using flow cytometry. The gating strategy for macrophages isolated from viable CD45^+^ mononuclear cells of human cervical cancer was shown in Fig. 2A. TAM was defined by CD45^+^CD14^+^CD11b^+^CD68^+^ and accounted for 3%-6% of all viable CD45^+^ leucocytes in cervical cancer. As was shown in Fig. 2B-E, the phenotype assessment showed that TAMs in cervical cancer tissue after radiotherapy were characterized by significantly decreased expression of CD163 (*p* = 0.0034) and significantly increased expression of the chemokine receptor CCR7 (*p* = 0.0015), indicating that the increased M1/M2 ratio of TAMs was present in cervical cancer after radiotherapy.

### No significant changes in phenotype of peripheral macrophages after radiotherapy

**Fig. 3:**
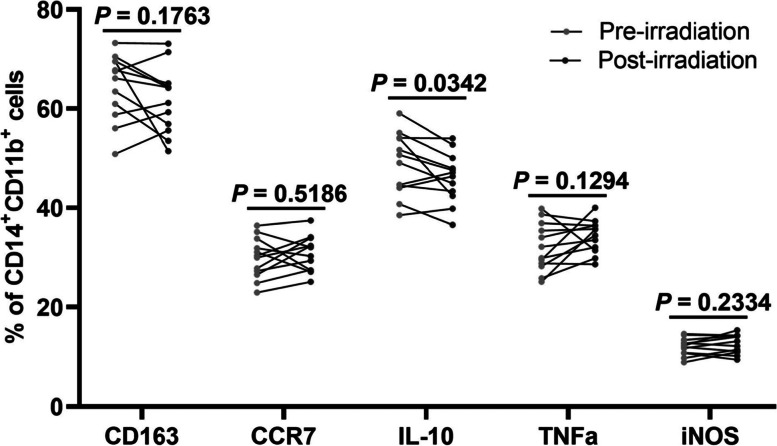
Flow cytometry analysis for peripheral blood mononuclear cells of cervical cancer patients. Peripheral blood mononuclear cells were obtained by Ficoll-Plaque density gradient centrifugation from cervical cancer patients before and after radiotherapy. The cell surface marker and intracellular cytokine were stained and analyzed using flow cytometry. *P* value was calculated by Wilcoxon matched-pairs signed rank test. IL, interleukin; TNFα, tumor necrosis factor-α; iNOS, inducible nitric oxide synthase

The phenotype of peripheral macrophages was also compared in cervical cancer patients before and after radiotherapy. The peripheral macrophages were defined as CD45^+^CD14^+^CD11b^+^ monocytes. As was shown in Fig. 3, except for a significant decrease in IL-10 expression, there was no significant change in expression level of CD163, CCR7, TNFα and iNOS of peripheral macrophages after radiotherapy. These results indicate that there was no significant change in polarization of peripheral macrophages in cervical cancer patients after radiotherapy.

### EVs from cervical cancer patients after radiotherapy contributes to the M2-like to M1-like phenotype transition

**Fig. 4:**
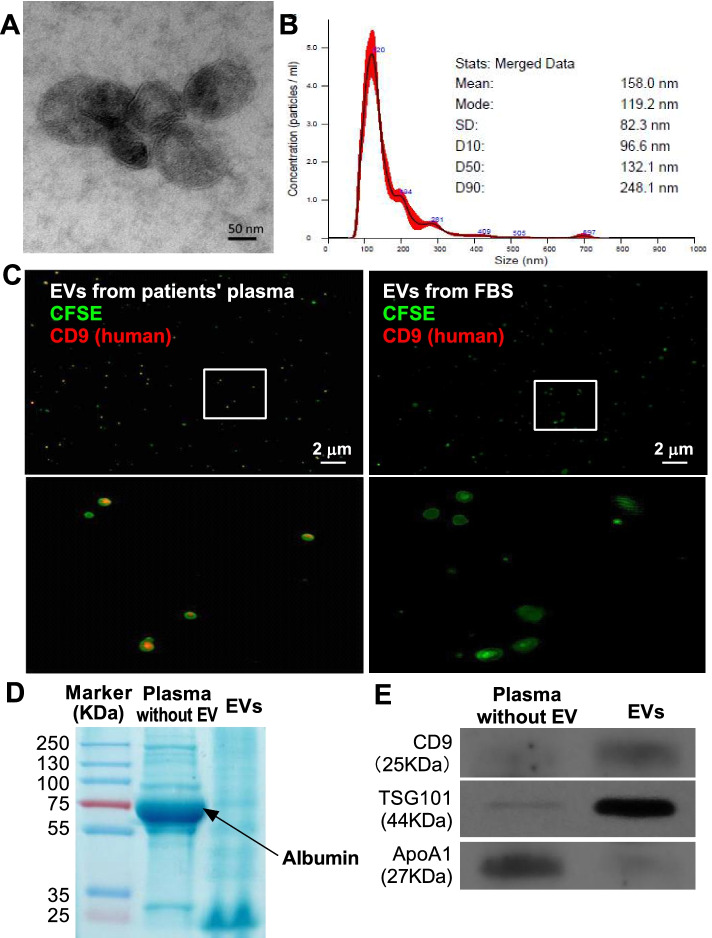
Identification of EVs isolated from the plasma of cervical cancer patients. The EVs were isolated from the fresh peripheral blood of cervical cancer patients using the Total Exosome Isolation Kit. **A**, a representative transmission electron microscopic image of EVs from plasma of post-irradiated patients was shown. EVs displayed with characteristic bilipid layer and size. **B**, representative nanoparticle tracking analysis of isolated EVs. **C**, immunofluorescence staining of characteristic EV marker (CD9) and membrane permeability to CFSE. The fluorescent stained EVs from fetal bovine serum (FBS) were also visualized as the negative control for anti-human CD9. EVs were visualized using a laser-scanning confocal microscope (TCS SP8 STED, Leica). The magnification of image below was 63×10. The total protein of EVs was separated by SDS-PAGE. D, Coomassie Blue staining was performed to demonstrate the significant differences in protein distribution. E, Western-blotting of EV markers (CD9 and TSG101) and non-EV marker (ApoA1) were performed. EV, extracellular vesicle; CFSE, carboxyfluorescein diacetate succinimidyl ester

**Fig. 5:**
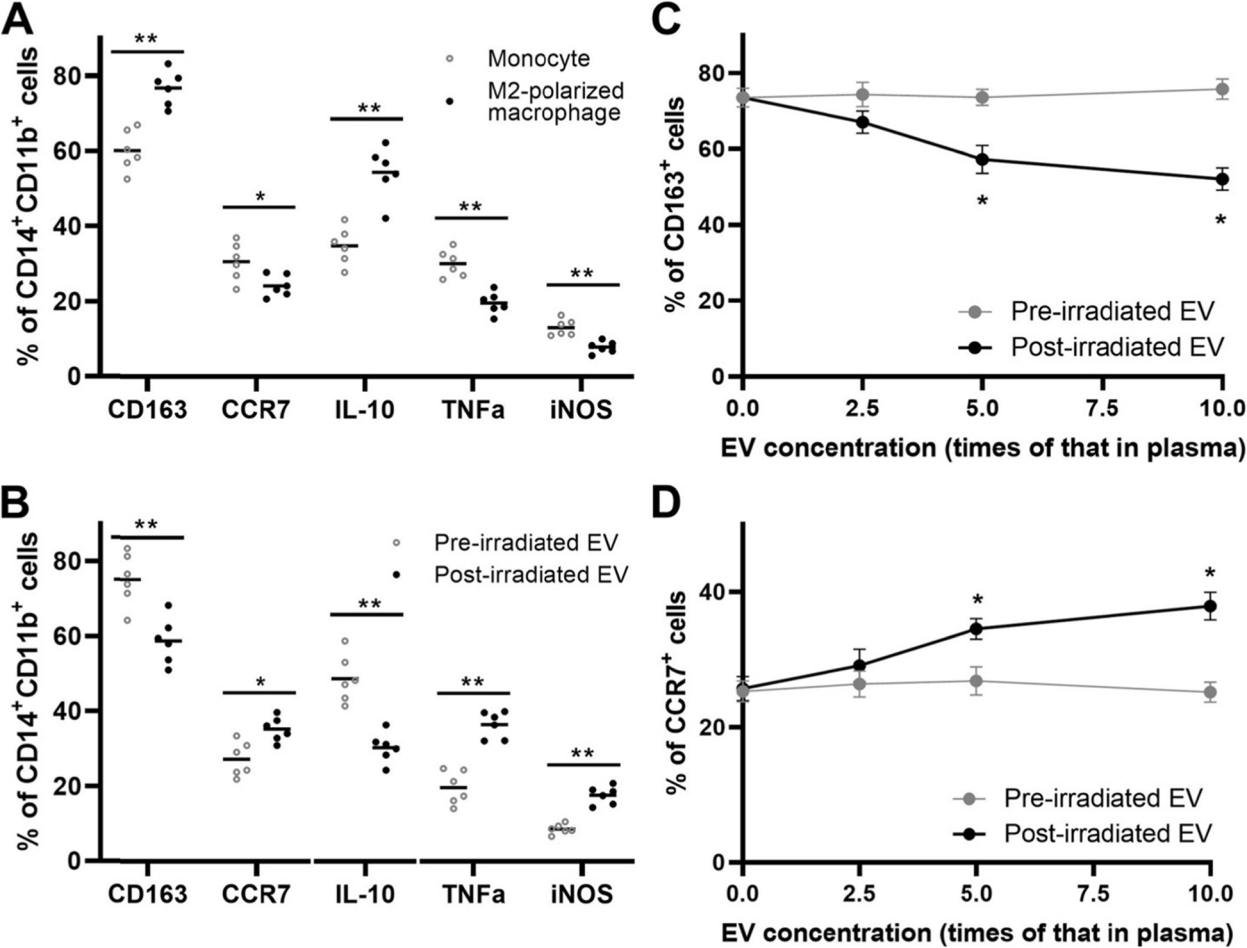
Polarization of macrophages can be facilitated by EVs from cervical cancer patients before and after radiotherapy. Peripheral blood mononuclear cells were obtained by Ficoll-Plaque density gradient centrifugation from healthy donors. Monocytes were isolated and cultured in RPMI 1640 medium. M2-polarized macrophages were obtained by IL-4+IL-13 stimulation for 48 hours. **A**, flow cytometry analysis the expression levels of cell surface and intracellular markers in macrophages before and after M2 polarization. M2 polarized macrophages were treated with EVs (the particle number per milliliter was 5 times higher than that in plasma) from cervical cancer patients before and after radiotherapy. **B**, flow cytometry analysis the expression levels of cell surface and intracellular markers. The dose effects of EVs on the expression levels of CD163 (**C**) and CCR7 (**D**) in M2 polarized macrophages were shown (n = 4). Bars presented as mean values of indicated markers. *, p < 0.05; **, p < 0.01. *P* value was calculated by two-tailed Mann Whitney U test

It has been reported that tumor-derived EVs demonstrated with immune-regulatory activities [16, 19]. Recent reports show that EVs from cancer patients can also promote macrophage polarization [25, 26]. To unravel the underlying mechanisms for TAM polarization in cervical cancer after radiotherapy, we compared the effect of EVs derived from cervical cancer patients before and after radiotherapy on polarization of macrophages from healthy donors. Blood EVs was isolated using the Total Exosome Isolation Kit (ThermoFisher Scientific). EVs were characterized by their distinct bilipid layer in electron microscopy (Fig. 4A), their size in nanoparticle tracking analysis (Fig. 4B), their positive expression of surface marker CD9 and the membrane permeability to CFSE in immunofluorescence staining (Fig. 4C), and their differential protein composition in Coomassie Blue staining (Fig. 4D) and Western-blotting analysis (Fig. 4E) compared with plasma protein. Considering that the TAMs demonstrated for increased M1-like polarization after radiotherapy, we first polarized peripheral blood monocyte-derived macrophages towards the M2-like phenotype. As was shown in Fig. 5A, the macrophages were polarized to M2-like phenotype after induction with IL-4+IL-13, characterized by significantly increased expression of CD163 (*p* = 0.0022) and IL-10 (*p* = 0.0022), and significantly decreased expression of CCR7 (*p* = 0.0411), TNFα (*p* = 0.0022) and inducible nitric oxide synthase (iNOS) (*p* = 0.0022). Interestingly, the polarization of these cells changed differently after exposure them to EVs of different origins. The M2-polarized macrophages exposed to EVs derived from cervical cancer patients after radiotherapy demonstrated for increased M1-like polarization compared with those before radiotherapy, characterized by significantly increased expression of CCR7 (*p* = 0.0108), TNFα (*p* = 0.0022) and iNOS (*p* = 0.0022), and significantly decreased expression of CD163 (*p* = 0.0043) and IL-10 (*p* = 0.0022), as was shown in Fig. 5B. To verify that higher doses of EVs (from patients after radiotherapy) indeed results in increased M1-like polarization, we investigated the effects of different concentrations of EVs on macrophage polarization. With the increase in EV concentration, the expression level of M2-like polarization related marker (CD163) decreased gradually (Fig. 5C), and M1 like polarization related marker (CCR7) increased gradually (Fig. 5D) in macrophages treated with EVs from patients after radiotherapy. However, there was no significant change in CD163 or CCR7 expression in macrophages treated with EVs from patients before radiotherapy. These results suggest that the role of EVs (from patients after radiotherapy) on macrophage polarization is dose-dependent.

### EVs from cervical cancer patients after radiotherapy contributes to increased phagocytosis

**Fig. 6:**
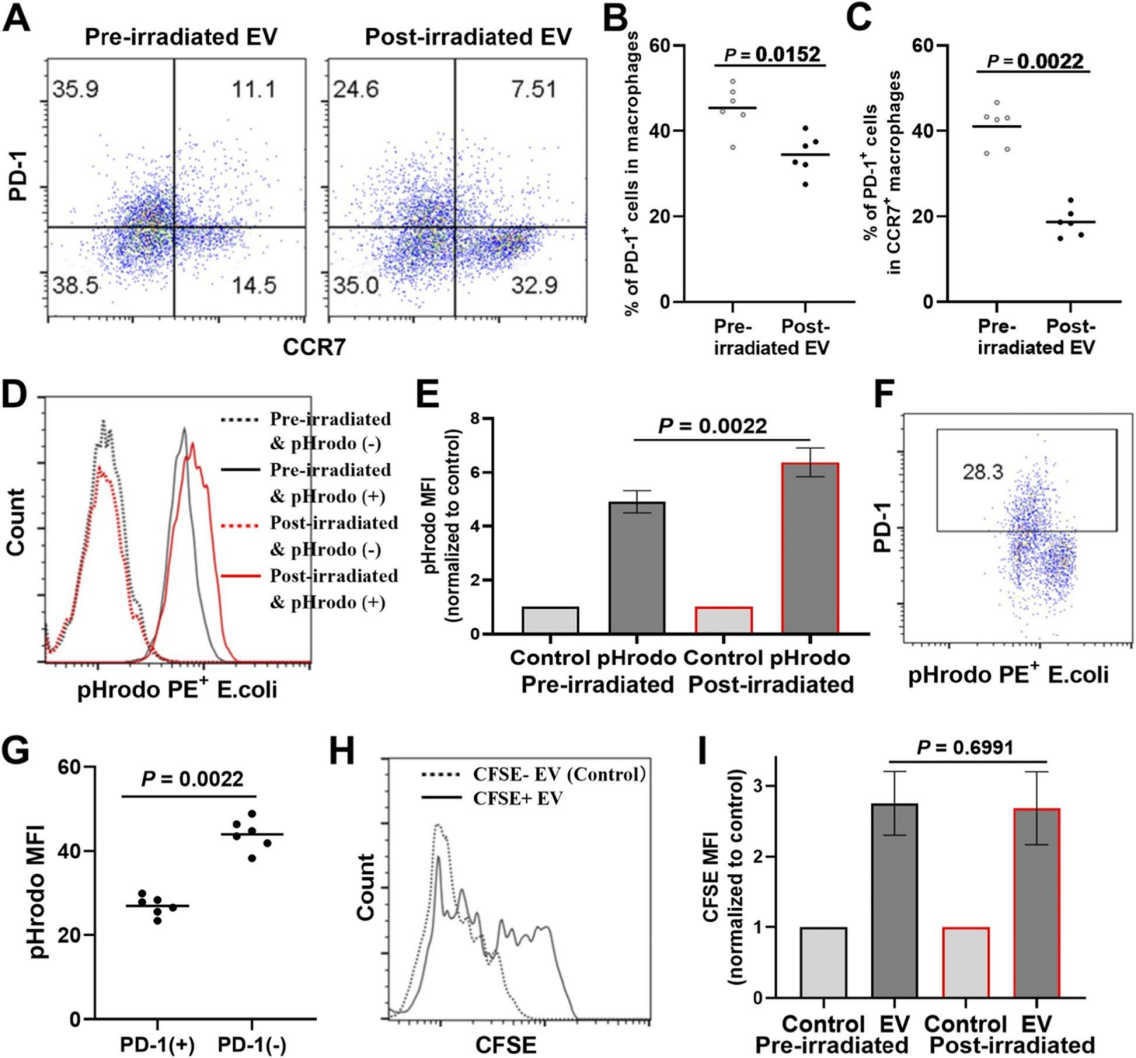
EVs from cervical cancer patients after radiotherapy contribute to increased macrophage phagocytosis. Peripheral blood mononuclear cells from healthy donors were obtained and cultured in RPMI 1640 medium. M2-polarized macrophages were obtained by IL-4+IL-13 stimulation and treated with EVs from cervical cancer patients before and after radiotherapy. **A**, representative scatter diagrams of flow cytometry analysis the expression of PD-1 and CCR7 in macrophages (CD45^+^CD14^+^CD11b^+^) were shown. Histograms of PD-1^+^ cells in total macrophages (**B**) and in CCR7^+^ macrophages (**C**) were shown. Macrophage phagocytosis was assessed using pHrodo Red E. coli bioparticles or CFSE labeled EVs. Representative diagram (**D**) and histogram (**E**) about mean fluorescent intensity (MFI) of pHrodo in macrophages were shown. Representative scatter diagram (**F**) and histogram (**G**) of flow cytometry analysis the correlation between PD-1 expression and MFI of pHrodo in macrophages were shown. Representative diagram (**H**) and histogram (**I**) of CFSE MFI in macrophages co-cultured with EVs labeled with or without (Control) CFSE, after treated with EVs from cervical cancer patients before and after radiotherapy for 24 hours, were shown. Data were presented as mean±SD. *P* value was calculated by two-tailed Mann Whitney U test

It was reported that the expression level of programmed cell death ligand-1(PD-1) in macrophages correlated with inhibited phagocytosis [27]. We then assessed the PD-1 expression of the macrophages exposed to EVs. As was shown in Fig. 6A-C, the M2-polarized macrophages exposed to EVs derived from cervical cancer patients after radiotherapy demonstrated for significantly decreased PD-1 expression compared with those exposed to EVs derived from cervical cancer patients before radiotherapy (*p* = 0.0152). More interestingly, there was a more significant decline in PD-1 expression (*p* = 0.0022) in CCR7^+^ macrophages between these two groups (Fig. 6C). These results indicate that, in addition to increased M1-like polarization, the phagocytic activity was also increased in macrophages exposed to EVs derived from cervical cancer patients after radiotherapy compared with those before radiotherapy. We then conducted the ex vivo phagocytosis assays using the pH sensitive PE-labeled E. coli particles, with increased fluorescence of E. coli particles indicating the phagosome. As was shown in Fig. 6D and E, after normalized to corresponding controls (absent for E. coli particles), the fluorescent intensity of macrophages exposed to EVs derived from cervical cancer patients after radiotherapy significantly higher than those before radiotherapy (*p* = 0.0022). More interestingly, the fluorescent intensity of PD-1 positive macrophages was significantly lower than that of negative ones (Fig. 6F and G), which also indicated the negative correlation between the phagocytic activity and the PD-1 expression of macrophages. We also assessed whether there was any difference in the phagocytosis of macrophage on EVs of different origin, using CFSE labeled EVs. The fluorescent of CFSE increased significantly after treated macrophages with CFSE labeled EVs than unlabeled EVs (control), as demonstrated in a representative example (Fig. 6H). There was no significant difference in phagocytosis of macrophage on EVs from patients before and after radiotherapy, as was shown in Fig. 6I. These results indicate that the role of EVs from different sources on macrophage polarization and phagocytosis may be due to the content of EVs that was devoured into macrophages, rather than the amount of EVs.

## Discussion

The present study demonstrate that radiotherapy of cervical cancer induces an increase in the number of TAMs and a change in their subtype from the M2-like phenotype to the M1-like phenotype. This clinical observation could be modeled through ex vivo stimulation of peripheral blood mononuclear cells from healthy donors with EVs from cervical cancer patients before and after radiotherapy, suggesting that EVs are important mediators in the changes of TAMs in cervical cancer patients caused by radiotherapy.

By inducing DNA strand break, radiotherapy can cause direct cell death. And hence, radiotherapy is an effective treatment for many kinds of cancers. Nevertheless, there may be more mechanisms in the radiotherapy of cancers. Among which, radiotherapy induced anti-tumor immunity being an attractive one [9–11]. As one of the abundant immune cell subsets in tumor tissue, macrophages had been reported to play roles in radiotherapy of cancers [28–30]. On the other hand, recent studies reported that tumor-derived EVs can modulate tumor-infiltrating immune cells, including TAMs, in multiple cancer types [16–19]. Therefore, it is interesting to clarify the role of tumor-derived EVs on change of TAMs induced by radiotherapy.

In our present study, we first demonstrated that there are increased TAMs in tissue of cervical cancer after radiotherapy. The further phenotypic assessment of the cell surface expression of CD163 and CCR7 indicated that TAMs tend to M1-like polarization after radiotherapy. In line with our results, there are reports that irradiation of cancer tissue promoted the expression of iNOS and then induced a pro-inflammatory phenotype of TAMs [31, 32]. It can be concluded that the M1-like polarization of TAMs is associated with radiotherapy. However, there was no significant change in expression level of CD163, CCR7, TNFα and iNOS in peripheral macrophages after radiotherapy, except for a significant decrease in IL-10 expression. The same results can be seen in the report by Pinto et al, that moderate doses of irradiation only caused decreased IL-10 expression as well as increased HLA-DR and CD86 expression in cultured peripheral macrophages, while other markers (CCR7, TNFα and IL-1B) were not altered [33]. These results indicate that the macrophage polarization induced by radiotherapy (the pelvic external-beam radiotherapy and brachytherapy) was limited to the irradiated cancer tissue. And in contrast, the effect of local radiotherapy on peripheral macrophages was not significant.

Considering the effect of tumor-derived EVs on macrophage polarization [19, 34, 35], we speculate that EVs play an important role in macrophage polarization induced by radiotherapy. To clarify whether the tumor-derived EV is one of the causes of phenotypic change of TAMs after radiotherapy, we treated the cultured macrophages with EVs derived from cervical cancer patients before and after radiotherapy. Our experiments demonstrate that EVs from cervical cancer patients after radiotherapy contributed to the M2-like to M1-like phenotype transition (increased expression of CCR7, TNFα and iNOS, and decreased expression of CD163 and IL-10). This phenotype transition is coupled with increased capacity of phagocytosis of TAMs (Fig. 5). Our results suggest that the change of macrophage polarization induced by radiotherapy in cervical cancer patients was at least partly mediated by EVs. We also demonstrate that there was no significant difference in the amount of EVs being devoured when the macrophage polarization had been changed by EVs of different origin, which is in line with a recent report by Stary et al [26]. These results indicate that the role of EVs on macrophage polarization and phagocytosis is due to the content of EVs, rather than the amount of EVs. However, we did not provide a candidate molecular through which cervical cancer derived EVs reprogrammed the macrophages to M1-like polarization. It has been reported that tumor derived EVs could regulate macrophage polarization through lncRNA [36], microRNA [37], protein [38], and so on. It is interesting to clarify the detailed mechanisms in irradiation induced TAM polarization of cervical cancer patients.

It was reported that expression of PD-1 inhibited the phagocytosis of TAMs [27]. Similar report can be seen in ex vivo irradiated rectal cancer tissue [26]. In line with these reports, we observed that the expression level of PD-1 in TAM of cervical cancer after radiotherapy was significantly decreased and, accordingly, the phagocytic activity of these TAMs was significant increased. More interestingly, there was a more significant decline in PD-1 expression of CCR7^+^ macrophages in our study. It has been reported that CCR7 not only was a marker for M1 macrophage [39, 40] but also was correlated with enhanced phagocytosis of antigens [41]. Therefore, PD-1 could be seen as a marker for immunosuppressive phenotype for macrophages.

In summary, our data demonstrated that irradiation in cervical cancer patients facilitated a proinflammatory macrophage phenotype which could eventually able to mediate anti-tumor immune responses. Our findings highlight the importance of EV in the crosstalk of tumor cells and TAM upon irradiation, potentially leading to an increased inflammatory response to cancer lesions.

## Supplementary Information

**Additional file 1: Supplementary Figure 1**: The original figure of Coomassie Blue stained gel. **Supplementary Figure 2**: The original Western-blot figure for CD9. **Supplementary Figure 3**: The original Western-blot figure for TSG101. **Supplementary Figure 4**: The original Western-blot figure for ApoA1.

## Data Availability

All data and materials generated or analyzed during this study are included in this manuscript.

## Abbreviations

EV: Extracellular vesicle
TAM: Tumor associated macrophage
CFSE: Carboxyfluorescein diacetate succinimidyl ester
iNOS: Inducible nitric oxide synthase
PD-1: Programmed cell death ligand-1
