# Supplementary material for: Extracellular vesicles mediated proinflammatory macrophage phenotype induced by radiotherapy in cervical cancer
Source: BMC Cancer. 2022 Jan 21;22:88. doi: 10.1186/s12885-022-09194-z (PMC8781113; doi:10.1186/s12885-022-09194-z)
Supplement: Supplementary file 1 — Additional file 1: Supplementary Figure 1: The original figure of Coomassie Blue stained gel. Supplementary Figure 2: The original Western-blot figure for CD9. Supplementary Figure 3: The original Western-blot figure for TSG101. Supplementary Figure 4: The original Western-blot figure for ApoA1. [file 12885_2022_9194_MOESM1_ESM.docx]

Supplementary Figure 1:  the original figure of Coomassie Blue stained gel.

Supplementary Figure 2:  the original Western-blot figure for CD9.


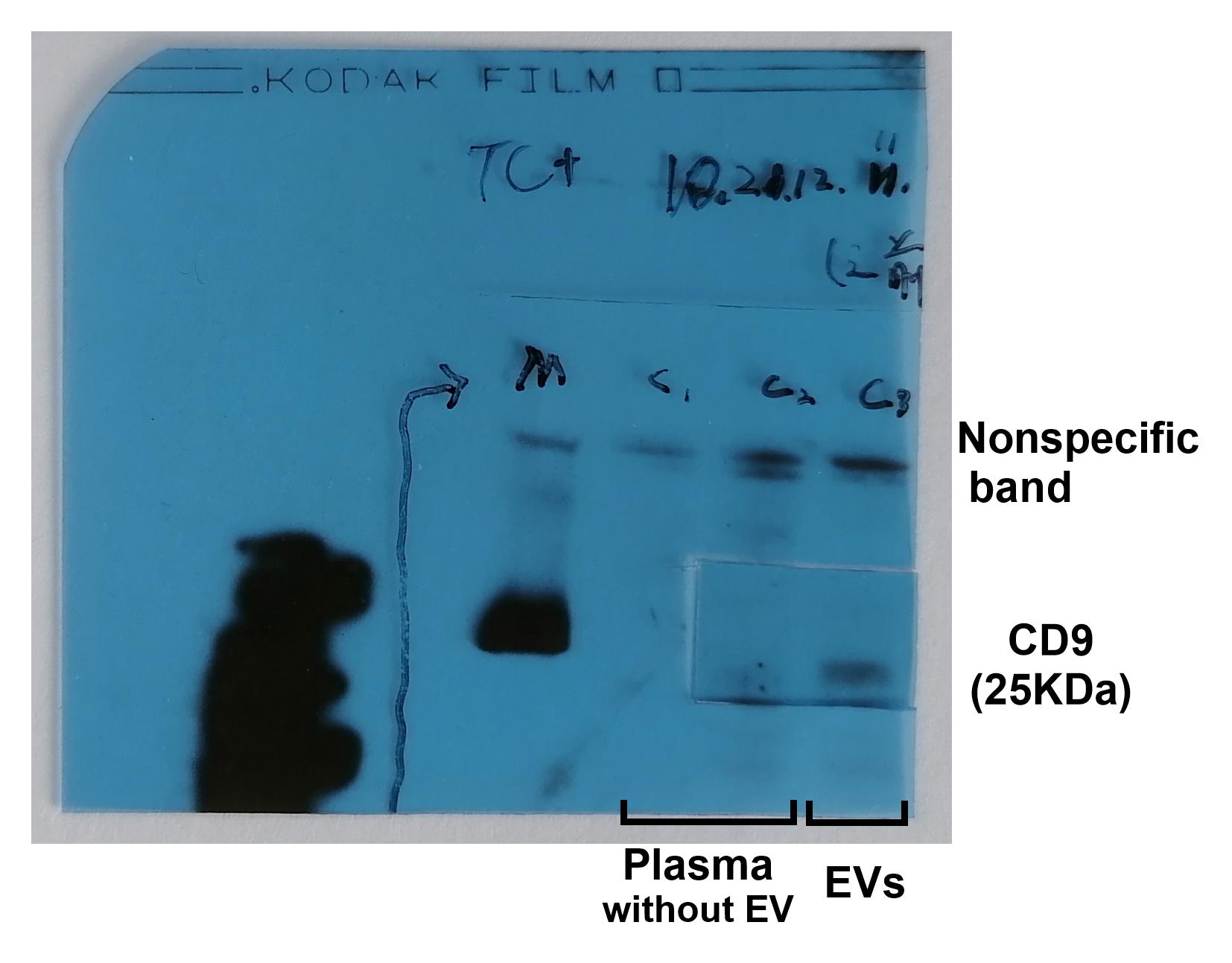


Supplementary Figure 3:  the original Western-blot figure for TSG101.


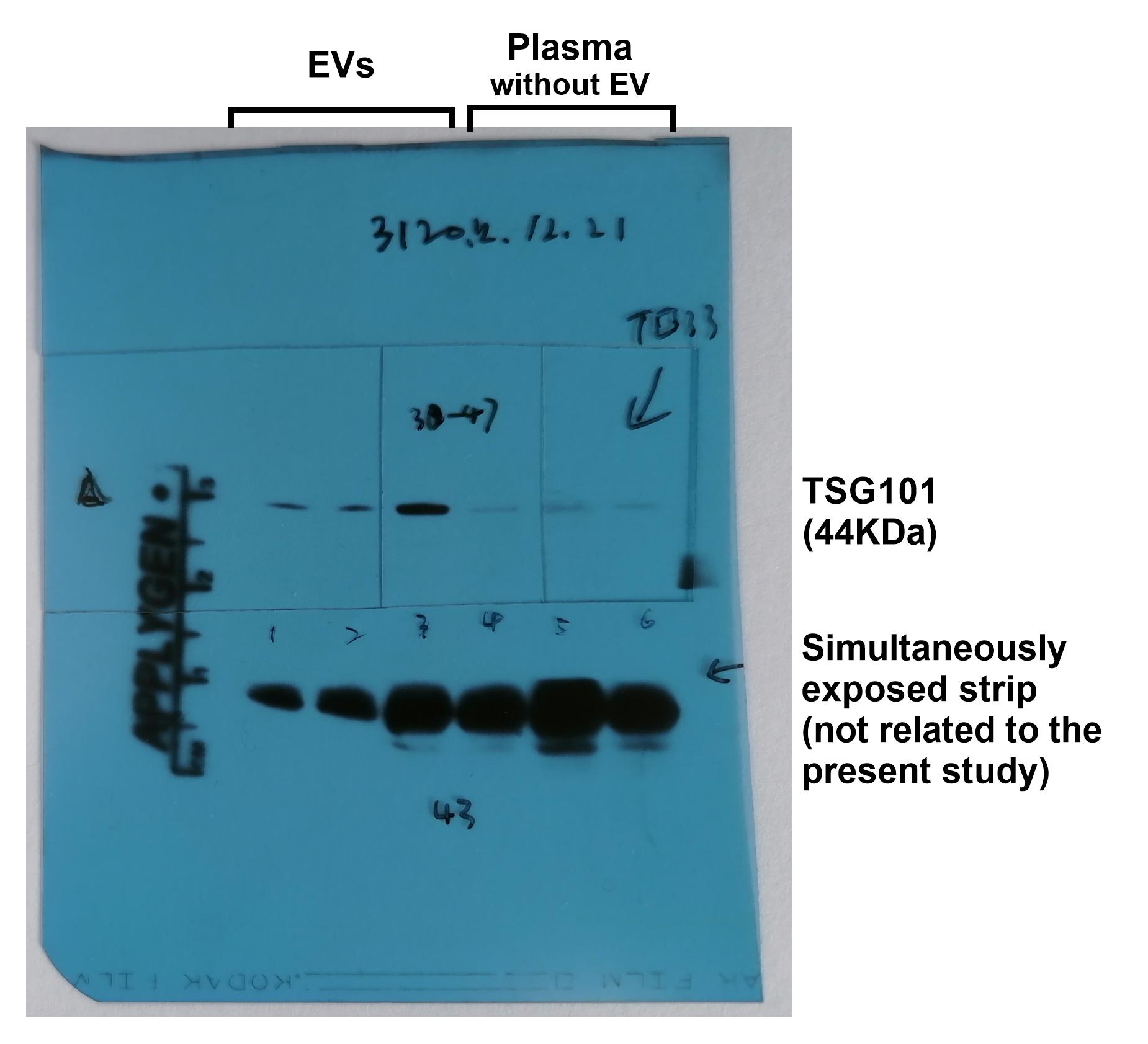


Supplementary Figure 4:  the original Western-blot figure for ApoA1.


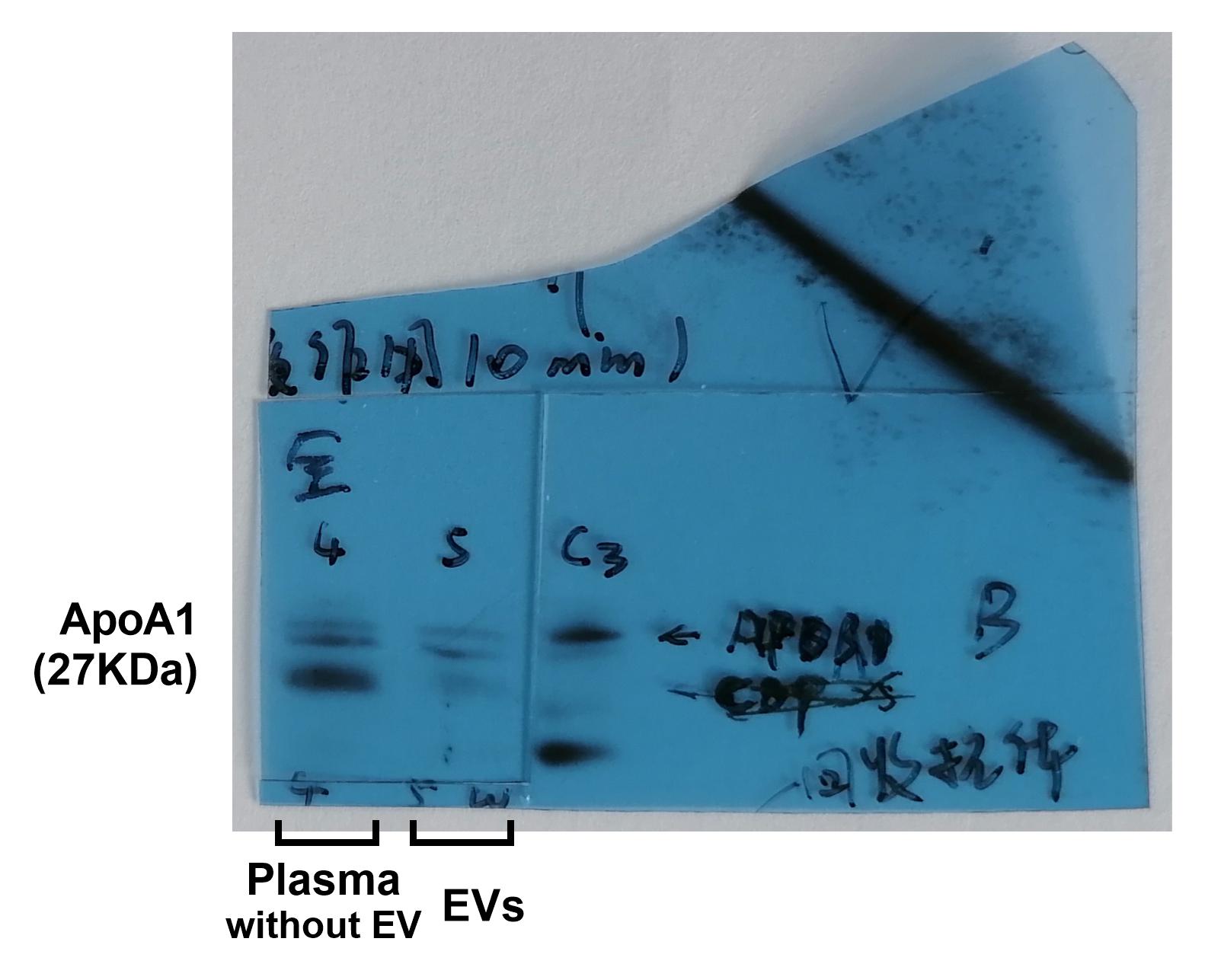


Supplementary Method:

Explanation for the absence of images of adequate length for Supplementary Figure 2-4.

Western-blotting for EV markers (CD9 and TSG101) and non-EV marker (ApoA1) were performed, according to our previously described methods [Int. J. Biol. Sci. 2014, 10(7):798-806]. In brief, EVs were lysed with RIPA lysis buffer and the protein concentration was determined using the BCA Protein Assay Kit. An equal amount of total protein (15 μg) from each sample was loaded. The protein suspensions were separated on 12% SDS-PAGE gel and transferred onto a nitrocellulose membrane. Prior to hybridisation with corresponding antibodies, the blots (nitrocellulose membrane) were cut into narrow strips according to the molecular weight of target proteins (And hence, the full-length blots can’t be obtained in the present study). The hybridized strips (containing luminescent solution) were exposed together with the film, and the final images were obtained.
